# Evaluating the Impact of Arginine-to-Lysine Ratios on Growth Performance, Antioxidant Defense, and Immune Modulation in Juvenile Largemouth Bass (*Micropterus salmoides*)

**DOI:** 10.3390/ani15131947

**Published:** 2025-07-02

**Authors:** Yulong Sun, Shuailiang Zhang, Xueyao Luan, Tao Liu, Jiale He, Jiteng Wang, Tao Han

**Affiliations:** 1 Department of Aquaculture, Zhejiang Ocean University, Zhoushan 316022, China; 2022214@zjou.edu.cn (Y.S.);; 2 Institute of Quality Standards and Testing Technology for Agricultural Products, Chinese Academy of Agricultural Science, Beijing 100081, China

**Keywords:** *Micropterus salmoides*, arginine, lysine, anti-oxidative, glutathione metabolism, inflammatory factors

## Abstract

This study examines the impact of the arginine/lysine ratio in feed on the growth, serum amino acids, arginine metabolism, and antioxidant capacity of juvenile largemouth bass (5.95 ± 0.02 g). Five isonitrogenous and isolipidic diets with varying arginine/lysine ratios were formulated and administered over an eight-week period. The results indicated that the treatments had no significant effect on protein efficiency ratio (PER), daily feed intake (DFI), or morphological indices of juvenile largemouth bass (*p* > 0.05). When the arginine/lysine ratio was 0.85 (2.25/2.65; 2.54/3.00), liver antioxidant capacity was maximized, and inflammatory factors were suppressed. Conversely, a ratio of 2.25/2.99 significantly reduced weight gain (WG) and specific growth rate (SGR) in juvenile largemouth bass, inhibited arginase activity, and increased serum total nitric oxide synthase (T-NOS) activity. When lysine was in excess (2.25/2.99 group), elevating arginine content (2.54/3.00 group) enhanced growth, antioxidant, and immune performance. Analysis of glutathione metabolism and innate immune-related pathway revealed that an optimal arginine/lysine ratio mitigates inflammatory damage induced by oxidative stress. An arginine/lysine imbalance significantly elevated liver malondialdehyde (MDA) content while reducing total antioxidant capacity (T-AOC), superoxide dismutase (SOD), catalase (CAT) activities, and glutathione (GSH) content, thereby increasing the expression levels of inflammatory factors (*IL1B*, *IL8*, *TGFB1*, *BAX*). These findings demonstrate that an imbalance in arginine/lysine adversely affects the growth, metabolism, and antioxidant capacity of largemouth bass. When lysine is in excess, increasing the arginine content to achieve an arginine/lysine ratio of 0.85 alleviates the negative effects of antagonism, suggesting arginine supplementation may regulate oxidative damage caused by lysine excess.

## Simple Summary

Optimal nutrition, particularly a balanced ratio of arginine to lysine, is critical for the healthy growth of juvenile largemouth bass. This study investigated the effects of varying dietary arginine-to-lysine ratios on growth performance, metabolism, and antioxidant capacity over an eight-week period. Results indicated that an arginine-to-lysine ratio of 0.85 enhanced hepatic antioxidant capacity and reduced inflammation. Conversely, excessive dietary lysine impaired growth, disrupted metabolic homeostasis, and increased oxidative stress. Supplementation with arginine under lysine-excess conditions improved growth, enhanced immunity, and bolstered antioxidant defenses, effectively mitigating the adverse effects of the amino acid imbalance. These findings suggest that maintaining an appropriate arginine-to-lysine ratio in aquaculture feed is essential for promoting growth and mitigating stress in juvenile largemouth bass, potentially leading to improvements in feed formulation.

## 1. Introduction

Nutritional immunology has gained global attention [1], particularly the interplay between essential amino acids and fish immune organs, along with their molecular mechanisms, which is a central focus in fish nutrition research [2,3,4]. Studies demonstrate that fish growth is closely linked to immune organ function, antimicrobial compounds [5], and the production of inflammatory factors [6]. Among essential amino acids, arginine and lysine are notable for their critical roles in animal nutrition. Beyond their function as protein synthesis substrates, these amino acids are involved in diverse metabolic activities and possess various physiological functions, influencing growth, development, metabolism, and health in fish [7,8]. Arginine, serving as a precursor for nitric oxide (NO), polyamines, creatine, and agmatine, has been identified as a key immunonutrient [9]. It is central to multiple metabolic pathways mediated by enzymes such as arginase, nitric oxide synthase, arginine: glycine amidinotransferase, and arginine decarboxylase [10], and its role in fish immunity has attracted widespread interest. Additionally, arginine and its metabolites contribute to cell proliferation, intestinal homeostasis, antioxidant capacity, and energy metabolism regulation [11,12], while also acting as potent stimulants for insulin and growth hormone secretion [13]. Conversely, lysine is the first limiting amino acid in many plant-based aquafeed protein sources. It functions as a precursor for carnitine, facilitating the transport of long-chain fatty acids to mitochondria for β-oxidation [14]. Lysine also regulates key metabolic and cellular signaling pathways, essential for nutrient utilization, growth promotion, and digestive system development in fish [15]. Its immune and antioxidant functions are well-documented [5,15,16,17], with optimal levels enhancing growth performance and intestinal antioxidant capacity [15,17]. However, lysine deficiency or excess can impair growth [18,19], increase oxidative stress [15], and exacerbate inflammatory responses [16].

Recent studies have extensively explored the requirements of arginine and lysine in different fish species [20,21,22]. These investigations consistently highlight the importance of maintaining appropriate amino acid levels for optimal growth, as deficiencies or excesses lead to reduced survival, stunted growth, and decreased feed efficiency [18,19,23,24,25,26]. However, most research has focused on individual amino acids, with limited investigation into their interactions. Arginine and lysine, both alkaline amino acids, share the same transport mechanism, leading to competitive uptake into cells [27,28]. Excessive lysine can reduce arginine availability [29], and their shared dibasic amino acid carrier suggests a potential antagonistic interaction that may influence absorption, transport, and metabolism [27,28,29]. While some studies have examined this interaction in aquatic species, results remain inconsistent [30,31]. For instance, Alam found no antagonism between arginine and lysine in olive flounder [30], whereas Dong observed significant antagonism in the giant freshwater prawn (*Macrobrachium rosenbergii*) [32]. Berge reported that high lysine levels inhibit arginine availability in Atlantic salmon muscle, though the specific mechanism remains unclear [33]. Thus, further research is required to comprehensively evaluate the impact of the interaction between dietary arginine and lysine on fish health. This evaluation should investigate the antagonistic effects of these amino acids on oxidative stress and inflammation from the perspective of nutritional immunology.

The largemouth bass (*Micropterus salmoides*), a freshwater carnivorous fish native to North America, was introduced to China in the 1980s and has since become a major aquaculture species due to its high-quality meat, short farming cycle, and adaptability. In 2023, largemouth bass farming in China exceeded 802,000 tons. While studies on the nutritional requirements of juvenile largemouth bass have been reported [34,35,36,37], gaps remain in understanding the interaction between arginine and lysine. Zhou et al. demonstrated that a diet containing 2.23% arginine optimizes growth performance [38]. Dairiki et al. suggested an optimal lysine supplementation level of 2.10% [39], while Coyle proposed that 2.80% lysine meets growth requirements [35]. However, no research has explored the potential antagonistic relationship between these amino acids. This study aims to investigate the effects of different arginine/lysine ratios on the growth performance, amino acid composition, antioxidant function, and arginine metabolism of juvenile largemouth bass, providing a theoretical basis for optimizing their inclusion in largemouth bass feed formulations.

## 2. Materials and Methods

### 2.1. Experimental Diets, Feeding Procedure, and Sampling

Diets were formulated using soybean meal, fish meal, and crystalline amino acids as primary protein sources, with fish oil as the fat source. All diets were iso-nitrogenous (45% crude protein) and iso-caloric (20 MJ·kg^−1^) (Table 1). The experiment comprised five treatment groups: D1, Optimal arginine-deficient lysine (Arg/Lys = 2.27/2.25); D2, Optimal arginine-optimal lysine (Arg/Lys = 2.25/2.65); D3, Optimal arginine-excess lysine (Arg/Lys = 2.25/2.99); D4, Excess arginine-excess lysine (Arg/Lys = 2.54/3.00); D5, Excess arginine-high lysine (Arg/Lys = 2.55/3.84).

Glycine was added to maintain iso-nitrogenity, and the amino acid composition mirrored the whole-body profile of juvenile largemouth bass (Table 2). Crystalline amino acids were weighed precisely and encapsulated using carboxymethyl cellulose (CMC) [35]. The pH of the mixture was adjusted to 7.0–7.5 with 6 N NaOH. The amino acid mixture was blended with dry ingredients, followed by the addition of oil and distilled water. The mixture was extruded into 1.5 mm pellets using a twin-screw extruder (G-250, South China University of Technology, Guangzhou, China). Pellets were dried at 50 °C for 12 h, cooled, and stored in sealed plastic bags at −20 °C until use.

Juvenile largemouth bass were obtained from Zhengda Aquaculture Co., Ltd., Huzhou, China and the experiment was conducted at the Nutrition and Feed Laboratory of Zhejiang Ocean University. After a 14-day acclimation period, 300 healthy fish (initial weight: 5.95 ± 0.02 g) were randomly allocated to 15 tanks, with five triplicate groups of 20 fish each. Fish were fed to satiation twice daily (8:30 and 17:30), and uneaten feed and feces were removed via siphoning 1 h post-feeding. Water temperature was maintained at 26 ± 1 °C, dissolved oxygen at >6 mg L^−1^, ammonia nitrogen at <0.05 mg L^−1^, and photoperiod at 12L:12D.

At the end of the 8-week trial, fish were fasted for 24 h and anesthetized with 150 mg L^−1^ MS-222. Fish were counted, weighed, and three individuals per tank were randomly selected for whole-body nutrient analysis. Blood was collected from six fish via the caudal vein, placed in 1.5 mL heparinized tubes, and stored at 4 °C for 12 h. Serum was isolated by centrifugation (4 °C, 4000 rpm, 10 min). Fish were dissected on ice, and viscerosomatic index (VSI), intraperitoneal fat ratio (IPF), hepatosomatic index (HSI), and condition factor (CF) were calculated. Tissue samples were flash-frozen in liquid nitrogen and stored at −80 °C for further analysis.

### 2.2. Sample Analysis

Proximate composition was analyzed according to AOAC standards [40]. Feed moisture was determined by drying at 105 °C for 24 h to constant weight. Whole fish and tissues were freeze-dried at −110 °C (LL1500, Thermo Scientific, Waltham, MA, USA) to constant weight for moisture content determination. Crude protein and crude fat were measured using an automatic Kjeldahl nitrogen analyzer (K355/K437, Büchi, Flawil, Switzerland) and Soxhlet extraction apparatus (E816, Büchi, Flawil, Switzerland), respectively. Ash content was determined by incineration at 550 °C for approximately 12 h. Total energy content of the feed and whole fish was measured using an adiabatic bomb calorimeter (HWR-15E, Shangli, Shanghai, China).

### 2.3. Biochemical Analysis and Amino Acid Determination

The activities of total nitric oxide synthase (T-NOS), aspartate aminotransferase (AST), alanine aminotransferase (ALT), total antioxidant capacity (T-AOC), superoxide dismutase (SOD), and catalase (CAT) were measured using commercial assay kits (Nanjing Jiancheng Bioengineering Institute, Nanjing, China). Serum ammonia, glutathione (GSH), and malondialdehyde (MDA) concentrations were determined following the manufacturer’s instructions, and absorbance was read using a microplate reader (Multiskan Go, Thermo Scientific, Waltham, MA, USA). Arginase activity was measured using a previously published method [41], with urea quantified via a colorimetric method using p-dimethylaminobenzaldehyde. One unit of arginase activity was defined as the amount of enzyme catalyzing the production of 1 mmol urea per minute.

To measure free amino acids in serum, 0.05 mL serum was mixed with 0.35 mL 8% sulfosalicylic acid. Then, 0.05 mL acetonitrile and 0.05 mL 2 M sodium bicarbonate were added, mixed thoroughly, and centrifuged (4 °C, 15,000 rpm, 10 min). The supernatant was collected for analysis.

For whole-body amino acids, 0.2 g of sample was hydrolyzed in 10 mL 6 N HCl under N_2_ for 10 min and incubated at 110 °C for 24 h. The sample was diluted to 100 mL with distilled water, and 0.5 mL supernatant was mixed with 0.5 mL acetonitrile, centrifuged (4 °C, 15,000 rpm, 10 min), and 0.2 mL supernatant was diluted with 0.8 mL 0.1 N HCl for amino acid analysis. All samples were analyzed using high-performance liquid chromatography (Agilent, 1260 Infinity II, Santa Clara, CA, USA) [42].

### 2.4. Determination of Gene Expression in Liver

Liver samples were collected from each treatment group post-trial. Fish were anesthetized with MS-222 (0.125 g/L), and blood was collected via the caudal vein using a 1 mL sterile syringe. Livers were dissected and flash-frozen in liquid nitrogen, and then stored at −80 °C for RNA extraction. Total RNA was isolated using TRIzol reagent (Invitrogen, Carlsbad, CA, USA), chloroform, isopropanol, ethanol, and DEPC water [43]. A total of 500 ng RNA was reverse transcribed into cDNA using the PrimeScript™ RT kit (Takara, Dalian, China) following the manufacturer’s instructions. Gene expression was analyzed using a Real-Time PCR system (QuantStudio™ 6 Flex, Life Technologies, Carlsbad, CA, USA), with relative expression levels calculated using the 2^−ΔΔCt^ method. Primer sequences are listed in Table S1 (Supplementary Materials).

### 2.5. Data Analysis

All data are expressed as mean ± SD. Statistical analyses were performed using SPSS (version 26.0). Data homogeneity and normality were assessed using Levene’s test and the Kolmogorov–Smirnov test, respectively. Significant differences were analyzed by one-way ANOVA followed by Tukey’s multiple comparison test (*p* < 0.05). Heatmaps were generated using R (v3.6.3) to visualize antioxidant and inflammation-related gene expression. Regulatory networks for the Keap1-Nrf2 signaling pathway and inflammatory factors were constructed using the GeneMANIA Cytoscape app (3.8.0). KEGG mapping results for glutathione pathway-related genes were generated using the OmicStudio tools platform (https://www.omicstudio.cn/tool/81) (accessed on 15 August 2024).

## 3. Results

### 3.1. Growth Performance and Morphological Indicators

The survival rate of largemouth bass was high (96.67–100.00%), with no significant differences among treatments (*p* > 0.05) (Table 3). The 2.25/2.65 group had the highest WG and SGR, while the 2.25/2.99 group had the lowest (*p* < 0.05). Although not statistically significant, the 2.54/3.00 group showed some improvement in WG and SGR compared to the 2.25/2.99 group. The highest FCR was recorded in the 2.27/2.25 group, although the difference was not statistically significant. Dietary treatments had no significant effect on PER, DFI, or FI (*p* > 0.05). Morphological indices were also unaffected by varying arginine/lysine ratios (*p* > 0.05) (Table 4). The CF in the 2.27/2.25 and 2.25/2.99 groups was numerically lower than in other groups.

### 3.2. Nutritional Components of the Whole Body and Tissues

The proximate composition of the whole fish, muscle, and liver was unaffected by dietary arginine/lysine ratios (*p* > 0.05) (Table 5). Liver fat content in the 2.25/2.99 group was numerically higher than in other groups.

### 3.3. Nitrogen, Lipid, and Energy Intake and Utilization

Daily lipid intake (DLI) was significantly affected by dietary treatments (*p* < 0.05) (Table 6). The DLI of the other four groups showed no significant difference compared to the control group (2.25/2.65) (*p* > 0.05). Although the difference was not statistically significant, DEI was highest in the 2.27/2.25 group.

### 3.4. Plasma Free Amino Acid Profile

The effects of dietary arginine/lysine ratios on serum amino acid composition are presented in Table 7. Among essential amino acids, Arg, Lys, and Met were significantly affected by dietary ratios (*p* < 0.05), while other essential amino acids remained unchanged. The 2.25/2.99 and 2.25/2.65 group exhibited significantly higher serum arginine content compared to other groups (*p* < 0.05). Serum lysine content increased with dietary lysine levels, except in the 2.54/3.00 group. Among non-essential amino acids, Asn, Gly, Ser, Tyr, and Cit were significantly influenced by dietary ratios (*p* < 0.05). Serum glycine content mirrored dietary glycine levels, while citrulline content in the 2.25/2.99 group was significantly higher than in other groups (*p* < 0.05). Moreover, the serum ornithine content in the 2.25/2.99 group achieved the highest numerical value (*p* > 0.05).

### 3.5. Whole Body Amino Acid Composition

Dietary arginine/lysine ratios had no significant effect on whole-body arginine and lysine content (*p* > 0.05; Table 8). However, His, Thr, Val, and Ser were significantly influenced by dietary treatments (*p* < 0.05). Amino acid retention rates indicated that Arg, Lys, and Gly retention rates decreased with increased dietary levels (*p* < 0.05; Table 9). Additionally, Ala, and Tyr retention rates were significantly impacted by dietary treatments (*p* < 0.05), with the lowest retention rates observed in the 2.27/2.25 group.

### 3.6. Serum and Liver Biochemical Indicators and Gene Expression Analysis

**Figure 1:**
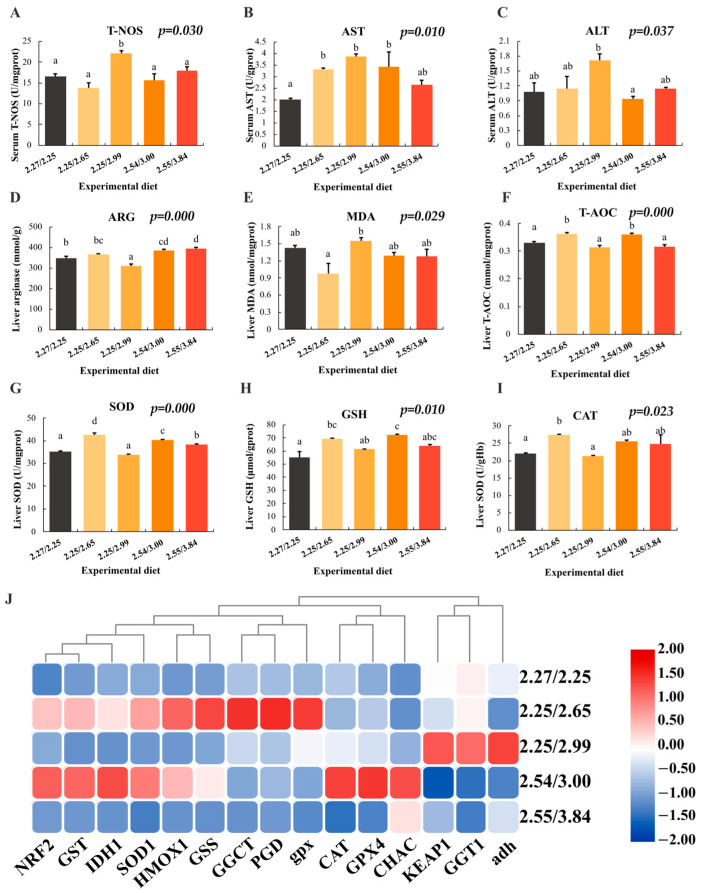
Serum and liver biochemical indicators and gene expression analysis under different arginine/lysine ratios. (**A**–**C**): Analysis of enzymatic activities of T-NOS, AST, and ALT in the serum of juvenile largemouth bass; (**D**): analysis of arginase activity in the liver of juvenile largemouth bass; (**E**–**I**): analysis of antioxidant-related indicators in the liver, including (**E**): MDA, (**F**): T-AOC, (**G**): SOD, (**H**): GSH, and (**I**): CAT; (**J**) heatmap of gene expression analysis related to the Keap1-Nrf2 pathway, antioxidant activity, and glutathione metabolism. Data are presented as mean ± standard deviation (SD) and were analyzed using one-way ANOVA. Different letters indicate statistically significant differences (*p* < 0.05) among groups.

Serum biochemical analysis revealed that T-NOS, AST, and ALT enzyme activities were highest in the 2.25/2.99 group (Figure 1A–C). Liver analysis showed that arginase activity in the 2.25/2.99 group (D3) was significantly lower than in the control group, while the 2.55/3.84 group (D5) exhibited significantly higher arginase activity (Figure 1D). Antioxidant-related indicators indicated that MDA content in the 2.25/2.99 group (D3) was significantly higher than in the control group, but decreased with increased dietary arginine levels in the 2.54/3.00 and 2.55/3.84 groups (Figure 1E). Liver T-AOC and SOD enzyme activities in the 2.25/2.65 and 2.54/3.00 groups (D2 and D4) were significantly higher than in other groups (Figure 1F,G). GSH content and CAT activity peaked in the 2.25/2.65 and 2.54/3.00 groups, respectively (Figure 1H,I).

qPCR analysis of liver antioxidant genes revealed that *SOD1*, *GST*, *HMOX1*, and *Nrf2* were significantly upregulated under optimal arginine/lysine ratios (D2 and D4 groups) compared to the control groups (D1). Meanwhile, *gpx* and *CAT* were upregulated in the D2 and D4 groups, respectively. *Keap1* exhibited a downward trend in the D4 group. Key glutathione metabolism-related genes, including *IDH1*, *GGCT*, and GSS, were significantly upregulated in the D2 and D4 groups. *PGD* and *GPX4* were upregulated in the D2 and D4 groups, respectively, while *GGT1* showed no significant differences. *CHAC* was upregulated in the D4 and D5 groups, potentially due to its involvement in multiple amino acid metabolic pathways (Figure 1J; Supplementary Materials, Figure S1).

**Figure 2:**
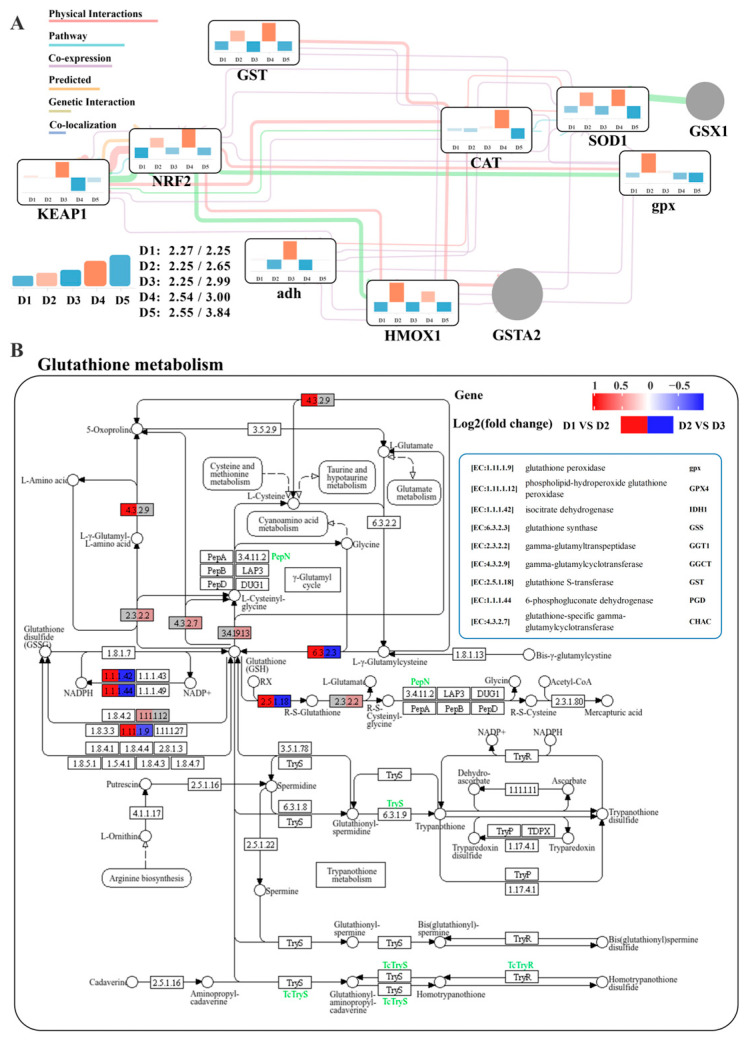
Integrated analysis of antioxidant and glutathione metabolism-related genes expression. (**A**): The interaction network of the Keap1-Nrf2 pathway and antioxidant-related genes represents the changes in the expression levels of key genes and their interactions under different arginine/lysine ratios. The color represents the relative expression value, where red and blue colors indicate up-regulation and down-regulation, respectively. The genes not subject to research are marked in gray. (**B**): KEGG mapping results of key genes in the glutathione metabolism pathway. The blue or red box illustrated significant downregulation or upregulation of the gene, respectively. White boxes or circles represent components not investigated in this study.

A molecular interaction network constructed using Cytoscape placed *Keap1* and *Nrf2* at the regulatory network’s starting point, controlling downstream genes such as *GST*, *CAT*, *adh*, *HMOX1*, *SOD1*, and *gpx* (Figure 2A). This highlights the critical role of the Keap1-Nrf2 pathway in regulating oxidative stress. Glutathione metabolism, crucial for the antioxidant system, was more active in the D2 group compared to the D1 group, with overall upregulated gene expression (Figure 2B). As the arginine/lysine ratio became imbalanced (D2 vs. D3), glutathione metabolism-related genes were downregulated, suppressing GSH synthesis and reducing antioxidant capacity.

**Figure 3:**
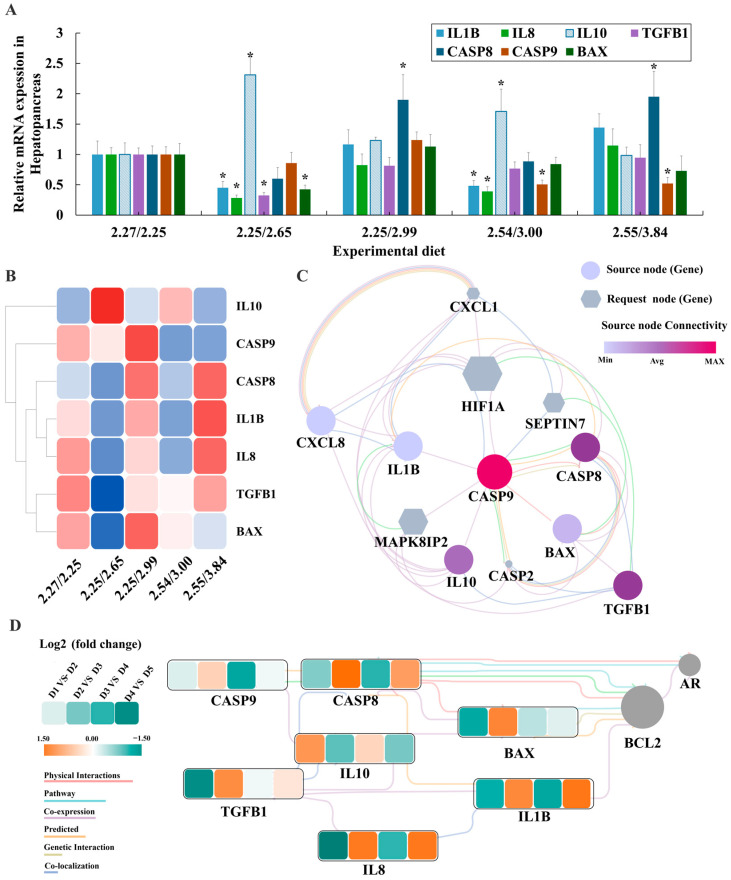
Integrated analysis of inflammation and apoptosis-related gene expression in the liver of juvenile largemouth bass. (**A**) Changes in the expression levels of inflammation and apoptosis-related genes under varying arginine/lysine ratios. Asterisks (*) indicate significant differences compared with the 2.27/2.25 group (*p* < 0.05). (**B**) Heatmap analysis of inflammation and apoptosis-related genes in the liver under different arginine/lysine ratios. The heatmap colors represent gene expression levels, with red, blue, and white indicating upregulated, downregulated, and unchanged expression, respectively. (**C**) Interaction network of inflammation and apoptosis-related genes, where purple circles represent the studied genes. The closer the color is to purple, the greater the gene’s importance in the network. Gray hexagons represent unstudied genes. (**D**) Interaction network of key inflammation and apoptosis-related genes based on qPCR results, illustrating changes in expression levels under different arginine/lysine ratios. Colors indicate relative expression values (Log^2^ fold change), with orange, green, and white representing upregulated, downregulated, and unchanged expression, respectively. Unstudied genes are marked in gray.

The antioxidant system’s interaction with inflammatory responses was evident, as antioxidants inhibited pro-inflammatory gene expression. Under optimal arginine/lysine ratios (D2 and D4 groups), pro-inflammatory genes *IL1B* and *IL8* were downregulated, while *TGFB1*, *BAX*, and *CASP9* were suppressed. Conversely, the anti-inflammatory gene *IL10* and apoptotic gene *CASP8* were upregulated as ratios shifted from optimal (D2 and D4 groups) to imbalanced (D3 and D5 groups) (Figure 3A,B). A Cytoscape regulatory network identified *CASP9* as the core gene, with *CXCL1*, *HIF1A*, *SEPTIN7*, *MAPK8IP2*, and *CASP2* as related genes (Figure 3C). Standardized analysis showed that most inflammatory factors (*CASP9*, *CASP8*, *IL1B*, *IL8*, *TGFB1*, *BAX*) were downregulated under optimal ratios (D1 vs. D2; D3 vs. D4), except for IL10 (Figure 3D). CASP9’s central position in the interaction network underscores its regulatory importance, with *BCL* and *AR* identified as additional key inflammatory genes.

## 4. Discussion

After an 8-week feeding trial, dietary treatments significantly influenced the growth performance of juvenile largemouth bass. The arginine and lysine levels in the D2 group were identified as optimal for this species. The highest weight gain (WG) and specific growth rate (SGR) values were observed in the D2 group, significantly surpassing those in the D3 group. Under the optimal dietary arginine content (D2, 2.25%; Arg: Lys ratio 0.85), both lysine deficiency (D1, 2.25%; Arg: Lys ratio 1.01) and excess (D3, 2.99%; Arg: Lys ratio 0.75) resulted in reduced growth performance and feed utilization in juvenile largemouth bass. These results further underscore the importance of maintaining optimal dietary levels of both arginine and lysine to promote growth in largemouth bass. The optimal Arg: Lys ratio for growth performance was determined to be 0.85. Deviations from this ratio, either an increase to 1.01 (D1) or a decrease to 0.75 (D2), resulted in reduced growth performance. These findings emphasize the need for precise dietary formulation to ensure balanced amino acid provision for optimal growth in largemouth bass aquaculture. Similar effects of imbalanced amino acid diets on growth performance have been reported in other species, including grass carp (*Ctenopharyngodon idella*) [17,44], golden pompano (*Trachinotus blochii*) [45] and yellow catfish (*Pelteobagrus vachelli*) [46].

Fish growth primarily depends on protein deposition, and a continuous supply of amino acids is essential for protein synthesis, as protein turnover is necessary for both growth and tissue repair. In this study, the D1 group showed the highest dry feed intake (DFI), dry nitrogen intake (DNI), and lysine retention rate, but the lowest nitrogen retention (NR) and protein efficiency ratio (PER) values. These findings suggest that lysine deficiency limits the utilization of amino acids for protein synthesis, thereby impeding the growth of largemouth bass. In agreement with this, Berge et al. [47] reported that Atlantic salmon fed a lysine-deficient diet exhibited poor FCR and PER. Ammonia is a major metabolic byproduct of undigested or unabsorbed proteins or amino acids in fish, and elevated blood ammonia levels can lead to toxicity or even death [48]. Studies on rainbow trout (*Salmo gairdneri*) [49], Atlantic salmon [47], black seabream (*Acanthopagrus schlegelii*) [50], and striped catfish (*Mystus nemurus* Cuv. & Val.) [51] have shown that dietary amino acid imbalances increase blood ammonia levels. It is generally believed that an imbalance between dietary arginine and lysine reduces protein turnover efficiency in fish, leading to increased amino acid oxidation for energy and elevated blood ammonia levels [52].

However, in this study, juvenile largemouth bass did not exhibit increased blood ammonia levels under imbalanced dietary arginine and lysine conditions. This may be because glutamate, glutamine, and aspartate supply 60–70% of the energy for largemouth bass [53], making energy metabolism less sensitive to imbalanced amino acids. Consequently, blood ammonia levels in juvenile largemouth bass were insensitive to the imbalanced dietary arginine and lysine levels.

On the other hand, increasing dietary lysine levels from 2.65% (D2 group) to 2.99% (D3 group) significantly reduced the growth performance of largemouth bass. Similar results have been reported in Japanese flounder [30]. This decline in growth performance may be attributed to the toxicity of excessive amino acids, which can hinder the absorption and utilization of other amino acids. Additionally, it could be due to the antagonistic interaction between arginine and lysine, resulting in more energy being expended on amino acid excretion rather than growth. Nutritional interactions between dietary amino acids, particularly imbalances and antagonisms, affect the efficiency of amino acid utilization for protein synthesis and other metabolic processes. Amino acid imbalance and antagonism are distinct concepts: imbalance can be corrected by supplementing the limiting amino acid, while antagonism can only be alleviated by supplementing the antagonized amino acid [54,55]. Although the D4 diet contained the same high lysine level (3.00%) as the D3 group (2.25/2.99; Arg: Lys ratio 0.75), the arginine level was increased to 2.54% (2.54/3.00) to replicate the Arg: Lys ratio (0.85) found in the D2 group. Notably, although not statistically significant, the D4 group showed a certain degree of improvement in growth performance compared to the D3 group. These results indicate that when excess lysine reduces the Arg: Lys ratio to 0.75 (D3, 2.25/2.99), subsequent arginine supplementation to restore the optimal ratio of 0.85 (D4, 2.54/3.00) can ameliorate growth performance declines resulting from amino acid imbalance, thereby mitigating antagonism. Similarly, Kim et al. reported that rainbow trout require higher dietary arginine levels when lysine levels are elevated [56]. Zhou et al. also found that supplementing arginine could reverse the negative effects of excess lysine on growth performance, feed efficiency, and liver arginase activity in black seabream [50]. Thus, increasing dietary arginine levels can partially mitigate the adverse effects of excessive dietary lysine, suggesting an antagonistic interaction between arginine and lysine in largemouth bass. Furthermore, juvenile largemouth bass fed the D5 diet (lysine 3.84%) did not show further improvement in growth performance. This may be due to increased deamination, leading to the excretion of excess lysine as ammonia, urea, or trimethylamine, resulting in amino acid wastage. Similar results were observed in olive flounder by Alam et al. [30]. In terrestrial animals, both arginine and lysine are basic amino acids that share the same transport mechanism, resulting in competition for transporters [27,28]. Excess lysine reduces the intracellular availability of arginine [29]. Iaccarino et al. [57] suggested that similar competition occurs in fish. Therefore, it is speculated that the antagonistic interaction between arginine and lysine in largemouth bass may also be influenced by transporter competition, although the exact mechanisms warrant further investigation. Specifically, the antagonistic effect between arginine and lysine should be evaluated comprehensively, taking into account feed intake, growth indices, digestion and absorption rates, and arginine metabolism parameters.

Free amino acids are critical for nutrient sensing and metabolic responses, with their concentrations influenced by dietary amino acid imbalances. In this study, serum lysine levels were strongly correlated with dietary lysine levels, increasing with dietary lysine content except in the D4 group. Although the difference was not statistically significant, serum lysine levels in the D4 group were substantially lower than those in the D3 group (147.26 vs. 90.35), suggesting that excess dietary arginine inhibits lysine deposition in largemouth bass. Consistent with this, Berge et al. [58] reported that excessive dietary arginine reduces plasma lysine levels in Atlantic salmon. Although the arginine content in the D4 and D5 group diets was significantly higher than in the first three groups, arginine retention rates in the D4 and D5 groups were considerably lower than in the D2 group, indicating that excess dietary arginine does not promote anabolic processes. Berge et al. [58] also noted that lysine can either stimulate or inhibit arginine absorption, depending on their relative concentrations. In this study, when dietary arginine was fixed at 2.25%, increasing dietary lysine significantly elevated serum arginine levels in juvenile largemouth bass. Additionally, increasing dietary lysine from 2.65% (D2 group) to 2.99% (D3 group) significantly raised serum citrulline levels. Previous studies on black seabream and zebrafish have shown that high dietary lysine levels inhibit arginase activity, reducing arginine metabolism efficiency. Similarly, in this study, increasing dietary lysine (2.99%) significantly reduced liver arginase activity in juvenile largemouth bass. Furthermore, T-NOS activity in the D3 group was significantly higher than in other groups. Since NOS and arginase compete for arginine in cellular metabolism [28], their relative activities determine arginine metabolite levels. These findings suggest that elevated dietary lysine decreases liver arginase activity, increases T-NOS activity, and elevates serum citrulline levels. Although not statistically significant, serum ornithine levels tended to increase with dietary lysine at both arginine levels. Berge et al. [47] also reported that increasing dietary lysine to 1.80% significantly elevated serum arginine and ornithine levels in Atlantic salmon. This may result from high lysine levels increasing T-NOS activity, promoting arginine metabolism into citrulline, and ultimately raising circulating ornithine due to enhanced arginase activity in largemouth bass. However, the effects of lysine on arginase activity vary among fish species. For example, increasing dietary lysine enhances tissue arginase activity in olive flounder [30], while no such effect was observed in yellow catfish (*Pelteobagrus fulvidraco*) [46].

ALT and AST are crucial aminotransferases present in the mitochondria of animal cells. These enzymes catalyze the transfer of amino groups from amino acids to α-keto acids and are released into the bloodstream following liver cell damage or injury [59]. In this study, serum ALT levels in the D3 group showed an upward trend compared to the other groups and were significantly higher than those in the D4 group. Serum AST levels also reached the highest value in the D3 group. Similar findings have been reported in black seabream [50] and blunt snout bream [60], where increasing dietary lysine levels under optimal arginine conditions led to elevated blood AST and ALT activities. These results suggest that an imbalance in dietary amino acids may disrupt amino acid metabolism in the liver, thereby impairing liver function. Fish antioxidant capacity, essential for self-defense and immune function, involves both enzymatic and non-enzymatic activities. MDA, a lipid peroxidation product, serves as a marker of oxidative damage [61]. T-AOC is a widely used measure of overall antioxidant activity, while SOD, CAT, and GSH are key antioxidant enzymes that form the primary defense against oxidative stress [62,63]. In this study, liver MDA levels in the D2 group were significantly lower than those in the D3 group. In contrast, T-AOC, SOD, GSH, and CAT activities in the D2 group were significantly higher than in the D1 and D3 groups. These findings indicate that lysine deficiency or excess compromises the antioxidant capacity of largemouth bass. Notably, T-AOC, SOD, and GSH activities in the D4 group (2.54/3.00) were significantly higher than in the D3 group (2.25/2.99), demonstrating that arginine supplementation mitigates oxidative damage induced by excessive lysine.

The Keap1-Nrf2 signaling pathway is critical for cellular antioxidant defense, regulating the expression and activity of antioxidant enzyme-dependent genes [64]. Glutathione, an endogenous antioxidant, plays a key role in preventing oxidative stress, scavenging free radicals, and reducing biological damage [65]. The Keap1-Nrf2 pathway controls genes involved in GSH synthesis and metabolism, such as *GS*, *GCL*, *GST*, and *GPx*, influencing de novo GSH synthesis and redox balance [66,67]. Additionally, Nrf2 promotes the transcription and translation of antioxidant genes, including *SOD*, *CAT*, *HMOX1*, and *NQO1*, which regulate cellular oxidative stress defense mechanisms [68,69,70]. Thus, activation of the Keap1-Nrf2 pathway is essential for enhancing GSH synthesis and the endogenous antioxidant response. In this study, *NRF2*, *SOD1*, *GST*, and *HMOX1* were significantly upregulated in the D2 and D4 groups under optimal arginine/lysine ratios, indicating that these ratios activate the Keap1-Nrf2 pathway to mitigate oxidative damage caused by excessive lysine. Furthermore, key glutathione metabolism genes were significantly upregulated in the D2 and D4 groups. Given the pivotal role of the Keap1-Nrf2 pathway in GSH homeostasis, it is hypothesized that this pathway activates GSH-dependent antioxidant systems to alleviate oxidative damage. The constructed glutathione metabolism regulatory network showed pathway suppression under imbalanced arginine/lysine ratios but upregulation under optimal conditions. Previous studies suggest that arginine mitigates oxidative stress and enhances immune function [10,71,72,73]. As a substrate for glutamate synthesis [74,75,76], arginine likely supports endogenous GSH synthesis [77]. Therefore, optimal arginine levels may exert antioxidant effects by modulating glutathione metabolism, though further targeted metabolic data are required for confirmation.

Excessive or deficient lysine impairs fish growth performance, increases oxidative stress damage [15], and exacerbates inflammatory responses [16]. Our analysis of key pro-inflammatory factors and the construction of a molecular regulatory network demonstrated that, under optimal arginine/lysine ratios (D2 and D4 groups), pro-inflammatory genes (*IL1B*, *IL8*, *TGFB1*, *BAX*) and apoptosis-related genes (*CASP9*, *CASP8*) were downregulated, while inflammatory factors increased as the arginine/lysine ratio shifted from optimal to imbalanced. In fish, the antioxidant system is closely linked to inflammatory responses [5], and antioxidants and antioxidant enzymes can suppress the expression of inflammatory genes, thereby maintaining health. Considering the observed activation of antioxidant genes and the glutathione metabolism pathway at the optimal arginine level in this study, we hypothesize that the optimal Arg: Lys ratio 0.85 may enhance the endogenous antioxidant system by activating glutathione metabolism, thus alleviating inflammatory responses. This further suggests that arginine supplementation may mitigate oxidative damage and inflammatory responses induced by lysine excess.

Although our findings indicate that an arginine-to-lysine ratio of 0.85 (2.54% arginine and 3.00% lysine) optimizes growth performance, antioxidant capacity, and immune responses in juvenile largemouth bass, certain limitations should be acknowledged. The eight-week experimental period may be insufficient to fully assess long-term physiological or metabolic adaptations; future studies with extended durations would help evaluate long-term impacts and nutritional adaptation mechanisms. Furthermore, regarding the antagonistic interaction between arginine and lysine, further assessment of arginine and lysine digestibility or amino acid transporter gene expression could strengthen our conclusions. While this study evaluated immunomodulation by focusing on immune and antioxidant marker expression, advanced techniques such as transcriptomics or proteomics could provide a more comprehensive understanding of immune regulation. Environmental factors (e.g., temperature, hypoxia) are also critical influences on animal nutritional regulation; future studies incorporating environmental stressors encountered in practical aquaculture could provide a more comprehensive and in-depth understanding, thereby enhancing the practical applicability of effective aquaculture feed formulations.

## 5. Conclusions

In conclusion, juvenile largemouth bass fed diets with 2.25% arginine and 2.65% lysine (Arg: Lys ratio 0.85) exhibited optimal growth performance. Imbalanced arginine/lysine ratios negatively affected growth, serum amino acid composition, antioxidant capacity, and immune responses. However, arginine supplementation partially mitigated the adverse effects of excessive lysine. Based on growth performance, serum amino acid composition, liver arginase activity, antioxidant enzyme activity, and inflammatory factor indicators, an antagonistic interaction between arginine and lysine was identified in juvenile largemouth bass. Furthermore, considering the activation of the antioxidant system and glutathione metabolism pathway, it is hypothesized that an optimal Arg: Lys ratio 0.85 may enhance the endogenous antioxidant system by activating glutathione metabolism, thus alleviating inflammatory responses. Increasing dietary arginine levels alleviated this antagonism when lysine was excessive, thereby mitigating oxidative damage and inflammatory responses induced by lysine excess.

## Figures and Tables

**Table 1:** Dietary formulation and proximate composition of the experimental diets (%dry matter).

Ingredients (g 100 g^−1^)	Argine/Lysine Ratio				
	D1: 1.01 (2.27/2.25)	D2: 0.85 (2.25/2.65)	D3: 0.75 (2.25/2.99)	D4: 0.85 (2.54/3.00)	D5: 0.66 (2.55/3.84)
Soybean meal	19.00	19.00	19.00	27.00	27.00
Fish meal	26.00	26.00	26.00	26.00	26.00
AA mix ^a^	15.22	15.22	15.22	11.64	11.64
Lys	0.00	0.51	1.11	0.86	1.96
Gly	2.81	2.29	1.67	1.13	0.00
Corn starch	12.00	12.00	12.00	12.00	12.00
Fish oil	9.92	9.92	9.92	9.92	9.92
Vitamin mix ^b^	1.10	1.10	1.10	1.10	1.10
Mineral mix ^c^	0.50	0.50	0.50	0.50	0.50
Calcium dihydrogen phosphate	1.00	1.00	1.00	1.00	1.00
Choline chloride	0.40	0.40	0.40	0.40	0.40
CMC	4.00	4.00	4.00	4.00	4.00
Cellulose	8.05	8.06	8.08	4.63	4.66
	100.00	100.00	100.00	100.00	100.00
Proximate composition (g 100 g^−1^ dry matter)					
Moisture	7.87	8.25	7.31	7.43	5.53
Crude protein	44.41	44.05	44.02	44.84	44.07
Crude lipid	12.26	12.32	12.32	12.05	12.61
Ash	8.74	8.96	9.08	9.06	9.50
Energy (MJ/kg)	20.57	20.46	20.60	21.16	21.48

^a^ Amino acid mixture (g kg^−1^ diet): Histidine, 1.6; Isoleucine 3.9; Leucine 24.0; Methionine, 5.5; Phenylalanine, 3.1; Threonine, 4.0; Tryptophan, 0.7; Valine, 4.9; Aspartic acid 18.1; Glutamic acid, 39.6; Serine, 6.6; Proline, 22.1; Glycine, 22.6; Alanine, 10.6; Tyrosine, 2.1; Cysteine 2.8. ^b^ Vitamin mixture (mg or IU kg^−1^ diet): Vitamin A, 10,000 IU; thiamine, 30; riboflavin, 60; Vitamin B6, 20; cyanocobalamin, 0.1; Vitamin C, 2000; Vitamin D3, 2000 IU; menadione, 40; Vitamin E, 160IU; Biotin, 2.5; Calcium pantothenate, 100; Folic acid, 10; Niacin, 200; Inositol, 100. ^c^ Mineral mixture (mg kg^−1^ diet): FeC_6_H_5_O7·5H_2_O, 13.2; MgSO_4_·7H_2_O, 53.2; KH_2_PO_4_, 93.2; NaH_2_PO_4_·2H_2_O, 33.2; AlCl_3_·6H_2_O, 2.8; ZnCl_2_, 8; CuSO_4_·5H_2_O, 4; MnSO_4_·H_2_O, 2.8; KI, 2.8; CoCl_2_·6H_2_O, 0.6; NaSeO_3_·H_2_O, 0.08.

**Table 2:** Amino acid composition of the experimental diets (%dry matter).

Amino Acid	Argine/Lysine Ratio				
	D1: 1.01 (2.27/2.25)	D2: 0.85 (2.25/2.65)	D3: 0.75 (2.25/2.99)	D4: 0.85 (2.54/3.00)	D5: 0.66 (2.55/3.84)
Arg	2.27	2.25	2.25	2.54	2.55
His	0.84	0.85	0.84	0.96	0.97
lle	1.63	1.67	1.59	1.64	1.67
Leu	2.82	2.84	2.79	2.94	2.96
Lys	2.25	2.65	2.99	3.00	3.84
Met	1.24	1.24	1.25	1.14	1.19
Thr	1.87	1.87	1.86	1.80	1.80
Phe	1.85	1.87	1.84	1.83	1.89
Val	2.40	2.37	2.33	2.28	2.27
Ala	3.15	3.15	3.12	3.07	3.07
Asp	5.22	5.23	5.21	5.16	5.19
Glu	9.10	9.10	9.05	9.00	9.02
Gly	6.60	6.14	5.53	5.00	3.94
Pro	0.55	0.56	0.54	0.54	0.57
Ser	2.01	2.00	2.03	2.13	2.15
Tyr	1.14	1.08	1.13	1.19	1.22

**Table 3:** Effect of arginine/lysine ratio on the growth performance of juvenile largemouth bass.

	Argine/Lysine Ratio					SEM ^1^	ANOVA ^2^
	D1: 1.01 (2.27/2.25)	D2: 0.85 (2.25/2.65)	D3: 0.75 (2.25/2.99)	D4: 0.85 (2.54/3.00)	D5: 0.66 (2.55/3.84)		*p* Value
Survival ^3^	98.33%	100.00%	100.00%	96.67%	100.00%	0.01	0.519
IBW ^4^	5.95 ± 0.10	5.92 ± 0.13	5.95 ± 0.06	5.98 ± 0.09	5.98 ± 0.06	0.02	0.930
FBW^5^	30.64 ± 0.86	32.41 ± 1.48	29.03 ± 0.59	32.21 ± 2.23	32.11 ± 1.36	0.46	0.068
WG ^6^	415.27 ± 5.95 ^ab^	447.29 ± 21.40 ^b^	388.34 ± 13.32 ^a^	438.53 ± 29.83 ^ab^	436.87 ± 23.23 ^ab^	7.23	0.033
SGR ^7^	3.15 ± 0.03 ^ab^	3.27 ± 0.08 ^b^	3.05 ± 0.06 ^a^	3.24 ± 0.11 ^ab^	3.23 ± 0.09 ^ab^	0.03	0.033
FCR ^8^	1.06 ± 0.04	0.98 ± 0.01	1.04 ± 0.03	0.97 ± 0.06	0.98 ± 0.03	0.01	0.035
PER ^9^	2.30 ± 0.08	2.52 ± 0.03	2.36 ± 0.07	2.48 ± 0.14	2.45 ± 0.06	0.03	0.053
DFI ^10^	2.76 ± 0.11	2.61 ± 0.03	2.64 ± 0.04	2.57 ± 0.13	2.58 ± 0.03	0.03	0.090

Data are expressed as mean ± standard deviation (n = 3). Different superscript letters (a, b) indicate significant differences among treatments (*p* < 0.05) The absence of superscript letters denotes no significant difference. ^1^ SEM: pooled standard error of means; ^2^ ANOVA: one-way analysis of variance, same as below. ^3^ Survival, % = 100 × final number/initial number. ^4^ Initial body weight, IBW, g. ^5^ Finial body weight, FBW, g. ^6^ Weight gain, WG, % = 100 × ((FBW − IBW)/IBW). ^7^ Specific growth rate, SGR, % day^−1^ = 100 × (ln (FBW) − ln (IBW))/day. ^8^ Feed conversion ratio, FCR = dry feed consumed/wet weight gain. ^9^ Protein efficiency ratio, PER = wet weight gain/protein intake. ^10^ Daily feed intake (DFI, g 100 g fish^−1^ day^−1^) = 100 × feed offered/average total weight/days.

**Table 4:** Effect of arginine/lysine ratio on the morphometrical parameters of juvenile largemouth bass.

	Argine/Lysine Ratio					SEM	ANOVA
	D1: 1.01 (2.27/2.25)	D2: 0.85 (2.25/2.65)	D3: 0.75 (2.25/2.99)	D4: 0.85 (2.54/3.00)	D5: 0.66 (2.55/3.84)		*p* Value
IPF ^1^	7.00 ± 0.33	7.37 ± 0.69	7.11 ± 0.18	6.87 ± 0.03	6.69 ± 0.03	0.10	0.115
HIS ^2^	1.49 ± 0.16	1.74 ± 0.32	1.53 ± 0.29	1.36 ± 0.06	1.36 ± 0.20	0.06	0.303
VSI ^3^	1.21 ± 0.16	1.34 ± 0.06	1.38 ± 0.03	1.39 ± 0.16	1.15 ± 0.07	0.03	0.073
CF ^4^	1.58 ± 0.27	1.87 ± 0.05	1.55 ± 0.31	1.84 ± 0.24	1.81 ± 0.03	0.06	0.109

^1^ Intraperitoneal fat ratio, IPF = 100 × (intraperitoneal fat weight/whole body weight). ^2^ Hepatosomatic index, HIS = 100 × (hepatosomatic weight/whole body weight). ^3^ Viscerosomatic index, VSI = 100 × (viscera weight/whole body weight). ^4^ Condition factor, CF = 100 × (live weight/length^3^).

**Table 5:** Effect of arginine/lysine ratio on the whole body and tissue proximate composition of juvenile largemouth bass.

	Argine/Lysine Ratio					SEM	ANOVA
	D1: 1.01 (2.27/2.25)	D2: 0.85 (2.25/2.65)	D3: 0.75 (2.25/2.99)	D4: 0.85 (2.54/3.00)	D5: 0.66 (2.55/3.84)		*p* Value
Whole body%							
Moisture	70.93 ± 1.88	71.56 ± 0.09	71.13 ± 1.03	72.14 ± 1.15	72.30 ± 0.11	0.28	0.212
Crude protein	17.01 ± 1.51	16.89 ± 0.23	17.22 ± 0.59	16.91 ± 0.41	17.17 ± 0.81	0.19	0.926
Crude lipid	8.46 ± 1.08	7.76 ± 0.38	8.18 ± 0.83	7.42 ± 0.99	6.70 ± 0.40	0.24	0.134
Ash	3.70 ± 0.36	3.65 ± 0.02	3.77 ± 0.07	3.41 ± 0.09	3.62 ± 0.14	0.05	0.220
Muscle%							
Moisture	76.55 ± 2.51	76.76 ± 0.22	73.21 ± 7.44	76.48 ± 0.62	76.69 ± 0.43	0.85	0.833
Crude protein	19.56 ± 1.08	19.58 ± 1.15	22.57 ± 6.14	19.09 ± 0.32	19.99 ± 0.46	0.70	0.600
Crude lipid	3.21 ± 1.76	3.23 ± 0.89	3.77 ± 1.75	3.34 ± 0.36	2.65 ± 0.27	0.28	0.621
Liver%							
Moisture	68.71 ± 0.77	70.06 ± 3.28	68.75 ± 3.54	69.90 ± 1.24	71.56 ± 1.52	0.59	0.585
Crude protein	10.70 ± 0.42	9.21 ± 1.31	10.88 ± 1.45	10.90 ± 1.24	10.78 ± 1.05	0.30	0.373
Crude lipid	3.33 ± 0.21	3.24 ± 0.30	4.10 ± 0.97	3.24 ± 0.19	3.17 ± 0.50	0.15	0.838

**Table 6:** Effect of arginine/lysine ratio on the nitrogen, lipid and energy utilization of juvenile largemouth bass.

	Argine/Lysine Ratio					SEM	ANOVA
	D1: 1.01 (2.27/2.25)	D2: 0.85 (2.25/2.65)	D3: 0.75 (2.25/2.99)	D4: 0.85 (2.54/3.00)	D5: 0.66 (2.55/3.84)		*p* Value
Nitrogen							
DNI (g kg^−1^ day^−1^) ^1^	1.81 ± 0.07	1.69 ± 0.02	1.72 ± 0.03	1.71 ± 0.09	1.72 ± 0.02	0.02	0.132
DNG (g kg ^−1^ day^−1^) ^2^	0.70 ± 0.08	0.71 ± 0.01	0.70 ± 0.04	0.71 ± 0.01	0.72 ± 0.04	0.01	0.834
NR (%) ^3^	38.74 ± 3.09	42.19 ± 0.29	40.48 ± 2.80	41.58 ± 2.86	41.93 ± 3.08	0.67	0.523
Lipid							
DLI (g kg ^−1^ day^−1^) ^4^	3.12 ± 0.12 ^a^	2.95 ± 0.03 ^ab^	3.01 ± 0.05 ^ab^	2.87 ± 0.15 ^b^	3.08 ± 0.04 ^a^	0.03	0.044
DLG (g kg ^−1^ day^−1^) ^5^	2.22 ± 0.36	2.04 ± 0.15	2.08 ± 0.29	1.91 ± 0.34	1.68 ± 0.10	0.08	0.217
LR (%) ^6^	70.90 ± 8.51	69.10 ± 4.94	69.19 ± 10.56	66.54 ± 8.45	54.59 ± 3.18	2.29	0.136
Energy							
DEI (10^2^ kJ kg^−1^ day^−1^) ^7^	5.20 ± 0.21	4.90 ± 0.05	5.00 ± 0.08	4.87 ± 0.26	4.99 ± 0.06	0.05	0.161
DEG (10^2^ kJ kg ^−1^ day^−1^) ^8^	2.05 ± 0.18	1.88 ± 0.23	1.86 ± 0.11	1.90 ± 0.20	1.94 ± 0.02	0.04	0.656
ER (%) ^9^	39.35 ± 1.86	38.39 ± 4.79	37.18 ± 2.77	38.94 ± 2.12	38.86 ± 0.82	0.64	0.827

Data are expressed as mean ± standard deviation (n = 3). Different superscript letters (a, b) indicate significant differences among treatments (*p* < 0.05) The absence of superscript letters denotes no significant difference. ^1^ Daily nitrogen intake; ^2^ daily nitrogen gain; ^3^ nitrogen retention; ^4^ daily lipid intake; ^5^ daily lipid gain; ^6^ lipid retention; ^7^ daily energy intake; ^8^ daily energy gain; ^9^ energy retention.

**Table 7:** Effect of arginine/lysine ratio on the serum amino acid composition of juvenile largemouth bass.

Amino Acid	Argine/Lysine Ratio					SEM	ANOVA
	D1: 1.01 (2.27/2.25)	D2: 0.85 (2.25/2.65)	D3: 0.75 (2.25/2.99)	D4: 0.85 (2.54/3.00)	D5: 0.66 (2.55/3.84)		*p* Value
Arg	2.96 ± 0.87 ^a^	5.29 ± 1.16 ^b^	6.73 ± 0.23 ^b^	2.97 ± 0.12 ^a^	2.16 ± 0.95 ^a^	0.49	0.028
His	70.22 ± 12.35	73.82 ± 11.65	101.46 ± 11.99	79.93 ± 17.46	104.28 ± 16.71	4.91	0.042
Ile	132.12 ± 30.69	139.91 ± 35.77	170.51 ± 14.73	146.28 ± 13.55	169.21 ± 14.65	6.66	0.248
Leu	227.08 ± 53.64	245.53 ± 58.98	299.12 ± 16.45	257.89 ± 28.41	297.29 ± 24.92	11.61	0.188
Lys	80.75 ± 35.35 ^a^	139.80 ± 31.71 ^ab^	147.26 ± 5.36 ^ab^	90.35 ± 18.10 ^a^	209.56 ± 46.78 ^b^	14.07	0.003
Met	77.33 ± 10.50 ^a^	82.35 ± 13.66 ^a^	120.80 ± 1.74 ^b^	95.59 ± 17.56 ^ab^	95.45 ± 21.61 ^ab^	5.14	0.035
Thr	206.75 ± 31.46	186.33 ± 40.69	252.06 ± 2.98	175.32 ± 41.32	225.96 ± 33.78	10.33	0.102
Phe	94.73 ± 23.25	96.60 ± 29.15	104.73 ± 17.76	103.79 ± 5.72	105.19 ± 18.93	4.62	0.946
Val	219.30 ± 48.31	230.31 ± 44.91	300.16 ± 21.31	246.26 ± 22.67	274.02 ± 24.26	10.89	0.087
EAA	1111.2467 ± 234.24	1199.94 ± 220.36	1502.83 ± 75.43	1198.39 ± 124	1483.13 ± 166.92	57.60	0.061
Ala	726.65 ± 178.62	693.38 ± 165.24	901.69 ± 37.56	667.97 ± 46.07	888.09 ± 95.40	37.26	0.103
Asp	23.90 ± 11.70	25.44 ± 1.49	26.71 ± 0.70	26.27 ± 2.07	28.01 ± 3.27	1.27	0.693
Asn	43.05 ± 16.60 ^a^	55.29 ± 5.92 ^ab^	87.41 ± 9.22 ^b^	61.20 ± 17.14 ^ab^	85.68 ± 9.66 ^b^	5.39	0.005
Glu	39.59 ± 13.75	46.58 ± 13.79	39.11 ± 4.53	35.48 ± 5.60	53.06 ± 8.82	2.77	0.287
Gln	213.62 ± 77.16	256.20 ± 80.53	365.19 ± 59.59	275.75 ± 51.72	248.46 ± 11.33	20.27	0.060
Gly	1880.44 ± 483.90 ^b^	1451.10 ± 245.11 ^ab^	1547.77 ± 110.18 ^ab^	1104.66 ± 253.94 ^a^	1116.80 ± 45.86 ^a^	97.78	0.036
Pro	83.42 ± 26.63	85.02 ± 25.21	100.46 ± 16.43	89.03 ± 2.56	115.26 ± 11.58	5.18	0.270
Ser	258.02 ± 58.75 ^ab^	282.89 ± 96.55 ^b^	304.09 ± 14.10 ^b^	134.23 ± 29.65 ^a^	136.82 ± 37.29 ^a^	22.95	0.032
Tyr	123.61 ± 19.47 ^ab^	108.69 ± 21.66 ^a^	175.84 ± 23.84 ^b^	140.32 ± 22.20 ^ab^	149.74 ± 30.02 ^ab^	8.02	0.049
Cit	44.59 ± 12.59 ^a^	42.96 ± 6.00 ^a^	74.76 ± 9.80 ^b^	49.65 ± 8.17 ^a^	45.54 ± 1.72 ^a^	3.67	0.005
Orn	78.52 ± 29.42	92.00 ± 23.10	120.25 ± 20.30	96.89 ± 1.56	109.71 ± 12.87	5.80	0.176
NEAA	3515.41 ± 861.72	3139.54 ± 653.80	3743.26 ± 215.71	2681.45 ± 392.72	3077.17 ± 144.54	151.43	0.201

Data are expressed as mean ± standard deviation (n = 3). Different superscript letters (a, b) indicate significant differences among treatments (*p* < 0.05) The absence of superscript letters denotes no significant difference.

**Table 8:** Effect of arginine/lysine ratio on the whole-body amino acid composition of juvenile largemouth bass.

Amino Acid	Argine/Lysine Ratio					SEM	ANOVA
	D1: 1.01 (2.27/2.25)	D2: 0.85 (2.25/2.65)	D3: 0.75 (2.25/2.99)	D4: 0.85 (2.54/3.00)	D5: 0.66 (2.55/3.84)		*p* Value
Arg	7.62 ± 0.13	7.58 ± 0.05	7.48 ± 0.02	7.52 ± 0.12	7.55 ± 0.16	0.03	0.573
His	2.72 ± 0.05 ^a^	2.73 ± 0.03 ^ab^	2.77 ± 0.02 ^abc^	2.82 ± 0.04 ^c^	2.82 ± 0.04 ^bc^	0.01	0.012
Ile	4.84 ± 0.11	4.90 ± 0.01	4.86 ± 0.06	4.79 ± 0.08	4.66 ± 0.15	0.03	0.064
Leu	8.17 ± 0.12	8.19 ± 0.09	8.30 ± 0.04	8.31 ± 0.13	8.27 ± 0.21	0.03	0.576
Lys	9.23 ± 0.19	9.37 ± 0.10	9.45 ± 0.07	9.51 ± 0.16	9.49 ± 0.22	0.04	0.269
Met	3.21 ± 0.07	3.27 ± 0.03	3.20 ± 0.09	3.22 ± 0.09	3.21 ± 0.06	0.02	0.792
Thr	4.94 ± 0.09 ^ab^	4.91 ± 0.04 ^a^	5.00 ± 0.04 ^ab^	5.03 ± 0.04 ^ab^	5.06 ± 0.04 ^b^	0.02	0.027
Phe	4.80 ± 0.05	4.81 ± 0.03	4.86 ± 0.01	4.85 ± 0.06	4.83 ± 0.07	0.01	0.466
Val	6.00 ± 0.05 ^b^	6.03 ± 0.02 ^b^	6.00 ± 0.02 ^b^	5.90 ± 0.09 ^ab^	5.76 ± 0.09 ^a^	0.03	0.003
EAA	51.52 ± 0.54	51.79 ± 0.27	51.92 ± 0.23	51.96 ± 0.42	51.64 ± 0.67	0.11	0.745
Ala	1.23 ± 0.02	1.21 ± 0.06	1.47 ± 0.04	1.32 ± 0.17	1.29 ± 0.05	0.03	0.071
Asp	10.84 ± 0.07	10.88 ± 0.05	10.92 ± 0.04	10.98 ± 0.13	10.99 ± 0.15	0.03	0.334
Glu	15.87 ± 0.12	15.86 ± 0.08	15.93 ± 0.06	15.98 ± 0.06	16.01 ± 0.10	0.02	0.205
Gly	10.81 ± 0.63	10.49 ± 0.34	10.06 ± 0.38	9.96 ± 0.70	9.97 ± 0.97	0.17	0.445
Pro	1.85 ± 0.10	1.91 ± 0.14	1.79 ± 0.05	1.77 ± 0.06	2.00 ± 0.15	0.03	0.135
Ser	4.85 ± 0.08 ^ab^	4.74 ± 0.07 ^a^	4.88 ± 0.06 ^ab^	4.92 ± 0.03 ^b^	4.99 ± 0.03 ^b^	0.03	0.004
Tyr	3.05 ± 0.12	3.12 ± 0.07	3.04 ± 0.20	3.12 ± 0.23	3.10 ± 0.20	0.04	0.963
NEAA	48.49 ± 0.54	48.21 ± 0.27	48.09 ± 0.22	48.05 ± 0.41	48.35 ± 0.67	0.11	0.741

Data are expressed as mean ± standard deviation (n = 3). Different superscript letters (a–c) indicate significant differences among treatments (*p* < 0.05) The absence of superscript letters denotes no significant difference.

**Table 9:** Effect of arginine/lysine ratio on the amino acid retention rate of juvenile largemouth bass.

Amino Acid	Argine/Lysine Ratio					SEM	ANOVA
	D1: 1.01 (2.27/2.25)	D2: 0.85 (2.25/2.65)	D3: 0.75 (2.25/2.99)	D4: 0.85 (2.54/3.00)	D5: 0.66 (2.55/3.84)		*p* Value
Arg	58.81 ± 3.44 ^ab^	64.25 ± 0.27 ^b^	59.99 ± 3.62 ^ab^	55.01 ± 3.62 ^a^	55.22 ± 3.46 ^a^	1.14	0.027
His	56.50 ± 3.31	61.46 ± 0.25	59.60 ± 3.59	54.72 ± 3.60	54.25 ± 3.40	1.01	0.070
Ile	52.21 ± 3.06	55.90 ± 0.23	55.05 ± 3.32	53.97 ± 3.55	52.07 ± 3.26	0.76	0.457
Leu	50.81 ± 2.97	55.14 ± 0.23	53.66 ± 3.23	52.38 ± 3.45	52.15 ± 3.27	0.74	0.468
Lys	72.09 ± 4.22 ^c^	67.46 ± 0.28 ^c^	57.10 ± 3.44 ^b^	58.79 ± 3.87 ^b^	46.16 ± 2.89 ^a^	2.50	0.000
Met	45.58 ± 2.67	50.41 ± 0.21	46.14 ± 2.78	52.31 ± 3.45	50.46 ± 3.16	0.92	0.047
Thr	46.26 ± 2.71	50.10 ± 0.21	48.41 ± 2.92	51.80 ± 3.41	52.50 ± 3.28	0.86	0.106
Phe	45.61 ± 2.67	49.08 ± 0.20	47.62 ± 2.87	49.23 ± 3.24	47.76 ± 2.99	0.67	0.492
Val	43.88 ± 2.57	48.60 ± 0.20	46.56 ± 2.81	48.02 ± 3.16	47.30 ± 2.97	0.71	0.268
EAA	52.67 ± 3.08	56.14 ± 0.23	52.81 ± 3.18	53.15 ± 3.50	50.38 ± 3.16	0.80	0.270
Ala	6.86 ± 0.41 ^a^	7.33 ± 0.03 ^a^	8.48 ± 0.51 ^b^	7.98 ± 0.53 ^ab^	7.84 ± 0.49 ^ab^	0.18	0.009
Asp	36.43 ± 2.13	39.74 ± 0.17	37.82 ± 2.28	39.44 ± 2.60	39.53 ± 2.48	0.58	0.316
Glu	30.62 ± 1.79	33.28 ± 0.13	31.75 ± 1.91	32.90 ± 2.17	33.13 ± 2.07	0.47	0.367
Gly	28.74 ± 1.69 ^a^	32.63 ± 0.13 ^ab^	32.80 ± 1.98 ^ab^	36.92 ± 2.43 ^b^	47.19 ± 2.95 ^c^	1.75	0.000
Pro	58.74 ± 3.44	65.54 ± 0.27	59.18 ± 3.57	60.32 ± 3.97	64.81 ± 4.06	1.06	0.090
Ser	42.37 ± 2.48	45.22 ± 0.19	43.33 ± 2.61	42.79 ± 2.82	43.34 ± 2.71	0.58	0.651
Tyr	47.08 ± 2.75 ^a^	55.09 ± 0.23 ^b^	48.48 ± 2.92 ^a^	48.62 ± 3.20 ^a^	47.49 ± 2.98 ^a^	0.97	0.023
NEAA	30.66 ± 1.80 ^a^	33.77 ± 0.14 ^ab^	32.58 ± 1.96 ^ab^	34.13 ± 2.25 ^ab^	35.86 ± 2.24 ^b^	0.61	0.059

Data are expressed as mean ± standard deviation (n = 3). Different superscript letters (a–c) indicate significant differences among treatments (*p* < 0.05) The absence of superscript letters denotes no significant difference.

## Data Availability

The data presented in this study are available upon request from the corresponding author.

## Supplementary Materials

The following supporting information can be downloaded at: https://www.mdpi.com/article/10.3390/ani15131947/s1, Figure S1: The expression levels of genes related to the Keap1-Nrf2 pathway (A), antioxidant mechanisms (A), and glutathione metabolism (B) in the livers of juvenile black bass were analyzed under varying ratios of arginine to lysine. Table S1: Primers used in this study.
