# Supplementary material for: Evaluating the Impact of Arginine-to-Lysine Ratios on Growth Performance, Antioxidant Defense, and Immune Modulation in Juvenile Largemouth Bass (Micropterus salmoides)
Source: Animals (Basel). 2025 Jul 2;15(13):1947. doi: 10.3390/ani15131947 (PMC12248939; doi:10.3390/ani15131947)
Supplement: Supplementary file 1 [file animals-15-01947-s001.zip › animals-3644383-supplementary.pdf]

Table S1. Primers used in this study.

Primer	Primmer sequencese	Primmer length (bp)	Amplification efficiency (%)	Accession number
IDH1-F	CGAGTCATCTGGGAGCTCATC	21	94.74	XM_038721367.1
IDH1-R	TTCTCATCTGGCGTGATGGTG	21		
PGD-F	GATCATGGCTTTGTGGTCTGC	21	90.22	XM_038739743.1
PGD-R	TGAAGTCATCCACAGCCTGTC	21		
GGCT-F	TCCAGCTCAAAAACCCCTCTG	21	93.38	XM_038710275.1
GGCT-R	TGTTCATCCTCCACACCACAC	21		
GPX4-F	TCAGCGACGGATATTGATGGC	21	99.4	XM_038716291.1
GPX4-R	TGGTTAGAAGGGAAGGCAAGG	21		
CHAC-F	AAACGCTGACATCCCAAACAC	21	102.16	XM_038726611.1
CHAC-R	TTAAAAATCCATGGCAGCCGC	21		
GGT1-F	TCAGCCGTGGATGCTTCTATC	21	100.06	XM_038704242.1
GGT1-R	CCAAACATGTCTTCAGTGGCG	21		
GSS-F	AGAAACCTGCTAACCTGACGG	21	94.94	XM_038723019.1
GSS-R	TGTGGGGAAGAGTGTGAATGG	21		
SOD1-F	GTGCAGGGCCTCATTTCAATC	21	97.57	XM_038708943.1
SOD1-R	TCTGCCAATGATCGAGTACGG	21		
CAT-F	AGCACAGAACTTCTCAGAGGC	21	104.45	XM_038704976.1
CAT-R	CCTTTTGCATGCACCACTCTC	21		
gpx-F	TCGTGAGAATGGCTGGGAATG	21	95.67	XM_038697220.1
gpx-R	ATAACGAGTCCCTTGGCAGTG	21		
GST-F	AAGGACATGACTCTGCTGTGG	21	97.69	XM_038724634.1
GST-R	CTGACCCCTGGGATTCATGTC	21		
adh-F	GCAGTGTTGGCAGCTTTAGAC	21	102.18	XM_038704220.1
adh-R	TCCTCTGGGTCATGTTTGGTG	21		
HMOX1-F	CCTGCAGAGATGGAGACCATG	21	93.1	XM_038694281.1
HMOX1-R	TTGTACTGTGGCAGGGTGATC	21		
NRF2-F	CCGCTCGCCATTTTAATCTGG	21	105.9	XM_038720536.1
NRF2-R	GCACCAAGATCAATGTCCTGC	21		
KEAP1-F	TTTGCAGAGTCATGTTGGTGC	21	98.23	XM_038728590.1
KEAP1-R	TGCTTTTACGCACGACTTGTC	21		
IL1B-F	CATCCAAAATCCCACTTGCGG	21	92.4	XM_038733429.1
IL1B-R	TACTGCTATTGTTGGCCAGGG	21		
IL8-F	CATGTCTGGGGGATCAAGGAG	21	90.43	XM_038713529.1
IL8-R	TTGACCCAAGGAGCATTAGGG	21		

IL10-F	CTCTTCCCGTCAGTCTCCTC	20	103.39	XM_038696252.1
IL10-R	ACCGAGGAGTCATTCTGGTG	20		
TGFB1-F	CACTTTGTCACCAGCAAGCTC	21	95.13	XM_038693206.1
TGFB1-R	TCACCTCGTTTATGCCCTCTG	21		
CASP8-F	ACACATCTGTTCAGTCCGACC	21	91.39	XM_038720546.1
CASP8-R	ACTCCACTGAGGTTGGGTTTC	21		
CASP9-F	GATTCTTCAGCGCAACAGGAC	21	101.23	XM_038723308.1
CASP9-R	GACTAACTGTCTGGCCTGGTC	21		
BAX-F	CATTTATCAGCGGGTTCAGCG	21	93.2	XM_038704178.1
BAX-R	TGGAGCTCTGCATTGCTATCC	21		
β-actin-F	GTGCCGTCTTCCCATCAATTG	21	102.58	XM_038703736.1
β-actin-R	TCCATGTCATCCCAGTTGGTG	21		

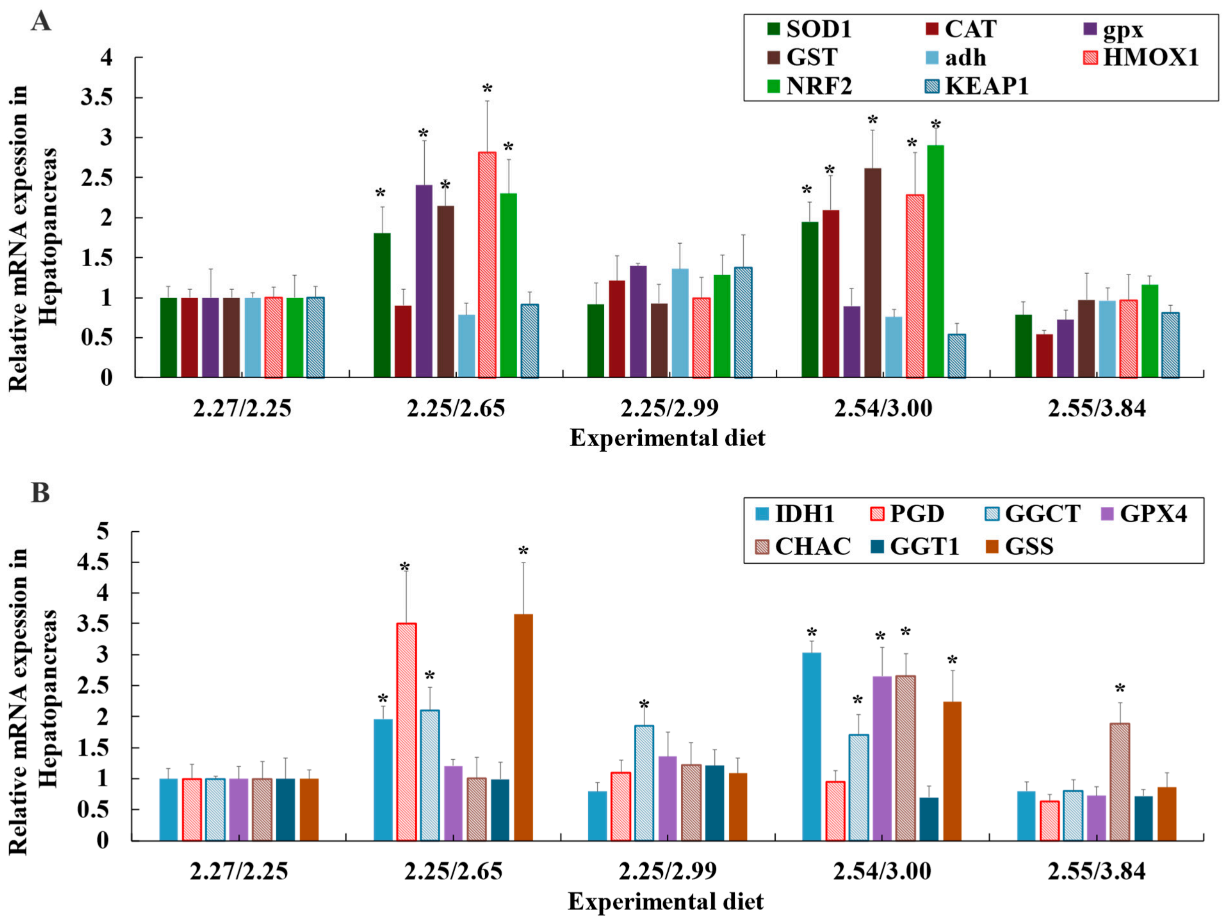

Figure S1. The expression levels of genes related to the Keap1-Nrf2 pathway (A), antioxidant mechanisms (A), and glutathione metabolism (B) in the livers of juvenile black bass were analyzed under varying ratios of arginine to lysine.
